# Synchronized Tactile Stimulation on Upper Limbs Using a Wearable Robot for Gait Assistance in Patients With Parkinson's Disease

**DOI:** 10.3389/frobt.2020.00010

**Published:** 2020-02-27

**Authors:** Takayuki Kishi, Taiki Ogata, Hiroki Ora, Ryo Shigeyama, Masayuki Nakayama, Masatoshi Seki, Satoshi Orimo, Yoshihiro Miyake

**Affiliations:** ^1^Department of Systems and Control Engineering, Tokyo Institute of Technology, Yokohama, Japan; ^2^Department of Computer Science, Tokyo Institute of Technology, Yokohama, Japan; ^3^Kikuchi Seisakusho Co. Ltd., Hachioji, Japan; ^4^WALK-MATE LAB Co. Ltd., Hachioji, Japan; ^5^Department of Neurology, Kanto Central Hospital, Setagaya, Japan

**Keywords:** gait assist, rhythm synchronization, stimulation on upper limbs, wearable robot, Parkinson's disease, abnormal gait

## Abstract

This study aimed to investigate whether using a wearable robot applying interactive rhythmic stimulation on the upper limbs of patients with Parkinson's disease (PD) could affect their gait. The wearable robot presented tactile stimuli on the patients' upper limbs, which was mutually synchronized with the swing of their upper limbs. We conducted an evaluation experiment with PD patients (*n* = 30, Modified Hoehn-Yahr = 1–3, on-state) to investigate the assistance effect by the robot and the immediate after-effect of intervention. The participants were instructed to walk 30 m under four different conditions: (1) not wearing the robot before the intervention (Pre-condition), (2) wearing the robot without the rhythm assistance (RwoA condition), (3) wearing the robot with rhythm assistance (RwA condition), and (4) not wearing the robot immediately after the intervention (Post-condition). These conditions were conducted in this order over a single day. The third condition was performed three times and the others, once. The arm swing amplitude, stride length, and velocity were increased in the RwA condition compared to the RwoA condition. The coefficient of variance (CV) of the stride duration was decreased in the RwA condition compared to the RwoA condition. These results revealed that the assistance by the robot increased the gait performance of PD patients. In addition, the stride length and velocity were increased and the stride duration CV was decreased in the Post-condition compared to the Pre-condition. These results show that the effect of robot assistance on the patient's gait remained immediately after the intervention. These findings suggest that synchronized rhythmic stimulation on the upper limbs could influence the gait of PD patients and that the robot may assist with gait rehabilitation in these patients.

## Introduction

Studies have shown that when two individuals walk side by side, their gait rhythms spontaneously synchronize with each other (Zivotofsky and Hausdorff, 2007). We focused on this mutual synchronizing phenomenon of gait rhythm to develop the Walk-Mate system to guide people's gait. The Walk-Mate system configures the mutual synchronization of human-human gait rhythms into a human-mechanical system. The Walk-Mate system uses a model of the mutual entrainment between non-linear oscillators, such as people's gait synchronized with rhythmic auditory cues generated by the system (Miyake, 2009). These auditory rhythmic cues that synchronize with people's gait could guide people's gait rhythm and reduce gait asymmetry (Miyake, 2009; Muto et al., 2012).

Parkinson's disease (PD) is one of many neurologic diseases characterized by an abnormal gait (Sveinbjornsdottir, 2016). PD is a progressive neurodegenerative disease in which the dopaminergic neurons in the substantia nigra pars compacta degenerate (Morris et al., 1994). In PD patients, gait rhythm is abnormal (Avanzino et al., 2016). This abnormal gait in PD patients is thought to be caused by the disruption of rhythm generation performed on the projection path entering the spinal cord via the basal ganglia and the brain stem and interferences of repeated rhythm movement (Freeman et al., 1993). Thus, rhythmic auditory stimulation has been used as a gait rehabilitation method for PD patients. One method is the rhythmic auditory stimulation (RAS) method in which a rhythmic sound with a constant tempo is presented to the patients. Studies have shown that gait improved when RAS was applied to patients with PD (Hausdorff et al., 2007; Thaut and Abiru, 2010). For example, RAS increased the stride length and velocity of the PD patients (McIntosh et al., 1997; Howe et al., 2003; Willems et al., 2006), and decreased the double support time (Freedland et al., 2002). The effects of RAS on PD patient's gait continued after the intervention (Thaut et al., 1996; Hausdorff et al., 2007). For instance, the increase in stride length and velocity were observed for 3 weeks (Thaut et al., 1996). These results show the effects of auditory stimulation and rehabilitation on gait assistance in PD patients.

Another method is auditory stimulation synchronizing with people's gait (Miyake, 2009; Muto et al., 2012). This auditory Walk-Mate system reduced the accelerated gait (Giladi et al., 2001) of PD patients, with the effect remaining immediately after the intervention (Uchitomi et al., 2016). Hove et al. investigated the effect of synchronized auditory cues on PD patient's gait from the viewpoint of the fractal scaling of stride times (Hove et al., 2012). The distribution of stride times in the gait of healthy people is not random; it has a 1/*f*-like structure, which represents a fractal-like long-range correlation of the stride times (Hausdorff et al., 1996; Hausdorff, 2009). That is, the fluctuations of their stride time are self-similar across multiple time scales. This self-similarity of the stride time is shown as log power proportional to log frequency in power analysis. The elderly with low fractal scaling of stride times have a higher risk of falling than those with high fractal scaling (Herman et al., 2005). In PD patients' gait, the fractal scaling of stride times is weaker than in healthy people (Hausdorff et al., 2000; Bartsch et al., 2007; Hausdorff, 2009). Hove et al. revealed that using the auditory Walk-Mate system, the 1/*f*-like structure in the stride duration distribution reappeared in PD patient's gait but not using RAS (Hove et al., 2012). In addition, the improvement of fluctuation seen in PD patients who used the auditory Walk-Mate system continued for several days (Uchitomi et al., 2013).

Yap et al. developed a wearable robot for gait assistance using the Walk-Mate system (Yap et al., 2019). This robot produces tactile stimuli on the upper limbs. The stimuli were synchronized with the swing of the upper limbs. The authors applied the robotic device to healthy elderly people and compared gait parameters of these individuals with and without wearing the robot. The results showed that the participants' hip swing angle increased in the gait while wearing the robot individuals. This result revealed that the synchronized tactile stimuli on the upper limbs improved people's gait performance via the coordination between the upper and lower limbs in walking (Dietz, 2002).

Although arm swing amplitude reduces, the coordination between the upper and lower limbs is preserved in PD patients (Dietz and Michel, 2008; Dietz, 2011). Therefore, if the upper-limb movements increased using the synchronized tactile stimulation on the upper limbs, the gait performance of the PD patients would increase. The purpose of this study was first to verify whether presenting synchronized tactile rhythm on the patient's upper limbs, as generated by the Walk-Mate robot, increased the gait performance of the patients with PD. As described in a previous study (Yap et al., 2019), we used tactile stimuli on the patient's upper limbs for two reasons. For evaluation of the robot, we used the following clinically important indexes: arm swing amplitude, stride length, velocity, and coefficient of variation (CV) of stride duration. A weak arm swing increases the fall risk of patients with PD (Wood et al., 2002). The smaller the stride length or velocity, the higher the risk of freezing (Hausdorff et al., 2003) of gait of patients with PD (Nieuwboer et al., 2001; Chee et al., 2009; Contreras and Grandas, 2012). The stride duration of CV is one index of gait rhythm stability. The larger the CV of stride duration, the higher the fall risk of patients with PD (Schaafsma et al., 2003). These three indexes were compared between gait with and without the presence of synchronized tactile stimuli with the patient's arm swing to reveal the functional effect of the robot's assistance on the patient's gate. As mentioned above, an increase of the stride length and velocity continued after the intervention by auditory rhythmic stimulation (Thaut et al., 1996; Hausdorff et al., 2007; Uchitomi et al., 2013). Thus, we compared these indexes between pre- and post-intervention measurements to reveal the immediate after-effect. The post-intervention trial was conducted several minutes after the trial with robot assistance. This post-intervention was performed to investigate the possibility of using the proposed system for rehabilitation in the future.

## Materials and Methods

### Algorithm to Present Rhythm Stimuli

**Figure 1:**
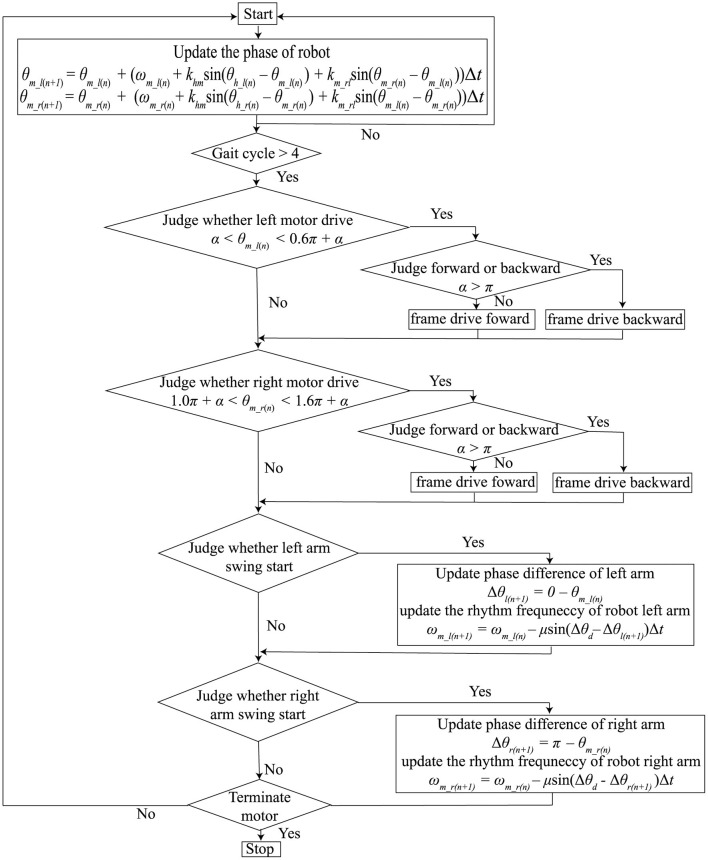
Flow chart of the core algorithm for presenting synchronized tactile stimuli with the patient's arm swing.

Figure 1 shows the flowchart of the core algorithm of the rhythm presenting system. The system comprises a rhythm mutual synchronization module, a phase difference control module, and a motor driving module. The first two modules are used for synchronizing the arm swings and tactile stimuli presented by the robot. For these modules, we used our previous method (Yap et al., 2019) based on the Walk-Mate model (Miyake, 2009), which assumed that each oscillator corresponding to both human arms and robot motors performed periodic motion. The motor driving module was used for controlling the motors equipped on the robot and presenting the tactile stimuli.

Mutual synchronization between the robot rhythm and human arm swing rhythm is determined by the two equations below:

$$\begin{array}{l} {\overset{\cdot }{\theta }}_{\text{m}\_\text{l}}=\omega_{\text{m}\_\text{l}}+{\text{k}}_{\text{hm}}\text{sin }\left(\theta_{\text{h}\_\text{l}}-\theta_{\text{m}\_\text{l}}\right)+{\text{k}}_{\text{m}\_\text{rl}}\text{sin }\left(\theta_{\text{m}\_\text{r}}-\theta_{\text{m}\_\text{l}}\right)\\ {\overset{\cdot }{\theta }}_{\text{m}\_\text{r}}=\omega_{\text{m}\_\text{r}}+{\text{k}}_{\text{hm}}\text{sin }\left(\theta_{\text{h}\_\text{r}}-\theta_{\text{m}\_\text{r}}\right)+{\text{k}}_{\text{m}\_\text{rl}}\text{sin }\left(\theta_{\text{m}\_\text{l}}-\theta_{\text{m}\_\text{r}}\right) \end{array}$$

where θ_*m*_*l*_ and θ_*m*_*r*_ represent the phases of the left and right robot motors. θ_*h*_*l*_ and θ_*h*_*r*_ represent the phases of the left and right arms. ω_*m*_*l*_ and ω_*m*_*r*_represent the angular frequencies of the left and right robot motors. *k*_*hm*_ represents the coupling strength between the left or right phases of the robot and human. We used the same coupling strength for the left and right coupling between the phases of the robot and humans. *k*_*m*_*rl*_ was the coupling strength between θ_*m*_*l*_ and θ_*m*_*r*_. θ_*h*_*l*_ and θ_*h*_*r*_ were not be able to be measured continuously. Therefore, we updated θ_*m*_*l*_ and θ_*m*_*r*_ only at the moment people's arms started swinging forward, as shown in Figure 1. The timing of the swing forward start was defined as the time from when the amount of change in the arm swing angle changed from a negative to a positive value. 0 rad and π rad were defined as the timing of the swing forward start of the left and right arms, respectively; θ_*m*_*l*_ and θ_*m*_*r*_ were updated if θ_*h*_*l*_ = 0 and θ_*h*_*r*_ = 0, respectively.

The phase different control module controlled the robot angular frequencies, ω_*m*_*l*_ and ω_*m*_*r*_, to make the phase differences between the robot and humans close to the pre-determined target value. Because the frequency of gait is different between individuals, the robot's frequencies should be modified; the robot can produce phase frequencies close to individual's frequency. In detail, the differentials of ω_*m*_*l*_ and ω_*m*_*r*_ were determined as follows:

$$\begin{array}{l} {\overset{\cdot }{\omega }}_{\text{m}\_\text{l}}=-\mu \text{sin}(\Delta \theta_{d}-(\theta_{\text{h}\_\text{l}}-\theta_{\text{m}\_\text{l}}))\\ {\overset{\cdot }{\omega }}_{\text{m}\_\text{r}}=-\mu \text{sin}(\Delta \theta_{d}-(\theta_{\text{h}\_\text{r}}-\theta_{\text{m}\_\text{r}})) \end{array}$$

where θ_*d*_ was the target value of the phase difference between the robot and humans. When θ_*d*_ = 0, the robot produced phase frequencies to match the individual's frequency. μ was the gain required to bring the pre-determined target value close to the phase difference values between the robot and humans. As in the rhythm mutual synchronization module, ω_*m*_*l*_ and ω_*m*_*r*_ were updated if θ_*h*_*l*_ = 0 and θ_*h*_*r*_ = 0, respectively.

In this study, we set *k*_*hm*_ = 0.25, *k*_*m*_*rl*_ = 2.5, μ = 0.08, and θ_*d*_ = 0. If the coupling strength between the robot and human, *k*_*hm*_, is over 1, the robot modifies the robot's phase more than the phase difference between the robot and human. This overcorrection destabilizes the robot's rhythm, which would also disturb human rhythm. If the coupling strength is under 0, the robot phase changes to increase the phase difference between the robot and human. Thus, the coupling strength should be between 0 and 1. The coupling strength between the left and right of the robot, *k*_*m*_*rl*_, was set much larger than *k*_*hm*_ to prevent the rhythm of the left and right tactile stimulation from being unstable by the phase correction using the phase difference between the robot and human. In human synchronization with external rhythmic stimuli, the correction of frequency is much weaker than that of the phase (Loehr et al., 2011). In addition, a rapid change of the robot's frequency would induce instability of the human's gait rhythm. Therefore, we set μ smaller than *k*_*hm*_. In synchronized auditory stimulation, the human's gait cycle could be guided faster or slower using θ_*d*_ (Miyake, 2009). In this study, to present the tactile stimuli at the exact timing we set, θ_*d*_ was set at 0.

**Figure 2:**
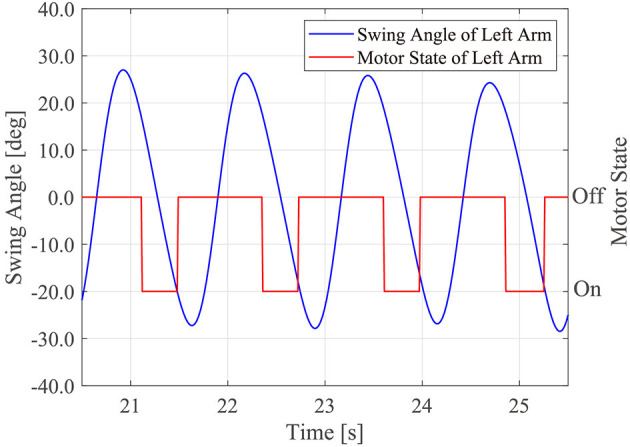
Sample time series of the human left arm swing angle and timing of the robot's motor driving.

The motor driving module controlled the timing of the motor driving using θ_*m*_*r*_ and θ_*m*_*r*_, which were calculated in the above two modules. The left motor was driven if α < θ_*m*_*l*_ < α + β and the right motor was driven if 1.0π + α < θ_*m*_*r*_ < 1.0π + α + β. α indicates the starting time of the motor. β is the duration of motor driving. If 0 < α < π, the tactile stimuli were presented while participants swung their arms forward. If π ≤ α < 2π, the tactile stimuli were presented while the participants swung their arms backward. Because the arm swing retroversion of the patients with PD was reduced (Roggendorf et al., 2012), we assumed that it was necessary to assist the backward arm swing of the patients with PD. Thus, α and β were set to 1.3π and 0.6π, respectively. That is, the motors were driven while the people swung their arm backwards from the timing at which the patients arm passed the side of the trunk backwards. Figure 2 shows the sample time series of the left arm swing angle and the robot's motor driving. Note that the motor driving module did not work in the first four gait cycles of the human after the robot first detects the human's gait phase because the robot phases were often unstable in the early stage of synchronization.

### Wearable Device

**Figure 3:**
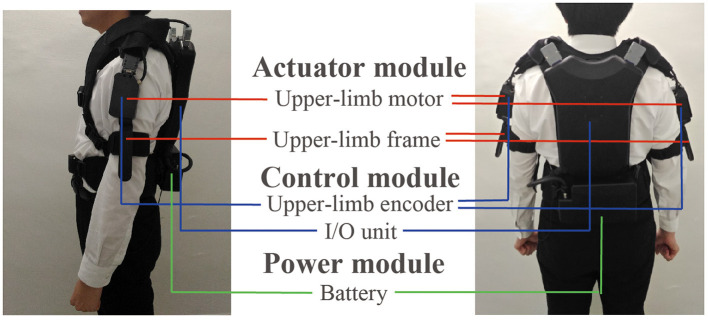
Appearance of the wearable robot and the three modules included in the robot: actuator, control, and power modules.

The wearable device comprised actuator, control, and power modules. Figure 3 shows the appearance of the device, which weighed 4.6 kg. The power module included the battery. The actuator and control modules are described below.

#### Actuator Module

DC brushless motors (DR-4316-X14B00421, ShinanoKenshi, Nagano, Japan) were mounted on the shoulder portions of the device. The motor torque to present rhythmic tactile stimuli was 0.074 kg. Because the weight of human's arm is several kg, the torque was not strong enough to mechanically move the patient's arms when the arms does not move. However, the torque would enhance the arm swing a little bit because the toque was generated in the same direction of the arm swing. The left and right frames were used to present the tactile stimuli. One of the two ends of the frame were fixed to the motor, and the other was fixed near the elbow by belts. The tactile stimuli were presented by rotating the frames through driving the motor.

#### Control Module

The control module comprised a microcomputer and encoders in the box placed on the back of the device. A microcomputer generated the robot rhythms and motor driving commands. The encoders were mounted on the left and right motors and measured the arm swing. The encoder measured the arm swing amplitude and the measurement range was ±150 degrees with reference to the position where the robot was turned on. The resolution was 1.2 degrees and the sampling frequency was 100 Hz. The swing angle data were measured to detect the timing of people's arm swing start for updating θ_*m*_*l*_, θ_*m*_*r*_, ω_*m*_*l*_, and ω_*m*_*r*_. The encoders were calibrated as the following before each trial. When the participants stood upright and their both arms straightened along their body, the robot was turned on. At that time, the angular of the encoders was set at 0.

### Experiment

#### Participants

The study group comprised 30 patients with PD (male, 18; female, 12). A prior power analysis (*f* = 0.25, α = 0.05, *power* = 0.9, *correlation among repeated measures* = 0.5) estimated the sample size to be 30. The mean age was 70.8 ± 8.8 years, the height was 163.9 ± 7.6 cm, and the weight was 58.7 ± 9.9 kg. The Modified Hoehn-Yahr severity stage (Goetz et al., 2004) ranged from 1 to 3. All participants were able to walk independently. All participants were idiopathic PD patients. They had taken medical treatment for PD and had been on-state before the experiment. We excluded the patients with freezing of gait or festination gait and those who needed walking assistance for gait. All participants did not suffer from musculo-skeletal or neurological pathologies, except for PD. This experiment was conducted with the approval from Tokyo Institute of Technology's Research Ethics Review Committee and Kanto Central Hospital Ethics Committee. Informed consent was obtained in writing from all participants.

### Task and Conditions

The experimental task involved walking 30 m on a flat floor. The task was conducted under four conditions: (1) pre-intervention condition, in which the participants walked without the robot before the intervention (Pre-condition); (2) robot-without-assistance condition, in which participants wore the robot and walked without rhythm assistance (RwoA condition); (3) robot-with-assistance condition, in which participants wore the robot and walked with the robot assistance (RwA condition); and (4) Post-intervention condition, in which the participants walked without the robot after the intervention (Post-condition). The RwoA condition was performed to eliminate the effect of wearing the robot. We compared the gaits between the RwoA and RwA conditions to evaluate the effect of gait assistance provided by the robot. We also compared the Pre- and Post-conditions to evaluate the immediate after-effect.

### Procedure

The participants began and stopped walking after receiving cues from the experimenter. We instructed participants to walk as usual, whether they wore the robot or not. In addition, the participants were asked to not synchronize with tactile stimuli from the robot under the RwA condition. It is well-known that the dual task decreases the gait performance of PD patients (Kelly et al., 2012; Raffegeau et al., 2019). When PD patients perform another task such as carrying a tray, generating words, and reacting to auditory of visual stimuli while walking, their stride length and velocity reduced. Therefore, we asked the participants not to consciously synchronize with the tactile stimuli. People unconsciously synchronize their movement to external stimuli (Zivotofsky and Hausdorff, 2007). Thus, we assumed that the participant's gait spontaneously synchronized to the tactile stimuli, even if they did not intend to synchronize their gait rhythm with the stimuli. The trials were conducted in the order of Pre-, RwoA, RwA, and Post- conditions. Under the Pre-, RwoA, and Post- conditions, the participants performed one trial. Under the RwA condition, the participants performed three trials. In the RwA condition, at the beginning of the trials, it took several strides to detect the participant's arm swing and for synchronization with the individual's gait rhythm. Thus, the number of the strides used for analysis was smaller in the RwA condition than the other conditions. Thus, we performed three trials with the RwA condition. Six trials were conducted with each participant. Between the trials, a break was taken for 1–2 min to prevent the effect of fatigue. It took ~40 min to complete the experiment. Two experimenters walked behind the participants in each trial to support them if they would fall down.

### Gait Analysis

We used the arm swing amplitude as the evaluation index to evaluate the effect of rhythm synchronization on the arm swing of patients with PD. The arm swings were measured by an encoder mounted on the robot. We defined arm swing amplitude as the absolute value of displacement of the arm swing angle in one cycle.

Stride length, velocity, and CV of stride duration were estimated using the IMU sensors to evaluate the lower limb movements (Hori et al., 2020). IMU sensors (TSND121, ATR-Promotions) were equipped on both of the participant's shanks. The IMU sensors measured three-axis acceleration and three-axis angular velocity. Measurement ranges were ± 8 G for the acceleration and ± 1,000 dps for the angular velocity; the sampling frequency was 100 Hz. Using the IMU sensor data, the trajectories of the shank and the timing of mid-stance were estimated; based on the trajectories, the stride length, velocity, and stride duration were calculated. This method was developed to measure the people's gait easily. The correlation was over 0.99 between the stride lengths estimated by the method and those measured by an optical motion capture system, though the method tended to slightly underestimate the stride length (Hori et al., 2020). The stride length was defined as the distance from one step to the next step in the anterior-posterior axis. The velocity was defined as the value obtained by dividing the stride length by the walking cycle in one cycle of gait. The stride duration CV was defined as the value obtained by dividing the standard deviation of the stride duration by the mean value of stride duration in one trial. We used the stride length and velocity as the evaluation indexes of the amplitude of the lower limb movement and stride duration CV as the evaluation index of gait rhythm stability.

### Statistical Analysis

We calculated the means of the indexes using the following method. Firstly, we calculated the mean value of each evaluation index on each limb for each trial. Secondly, we calculated the mean value of both limbs for each trial. Finally, we calculated the mean value of the three trials of the robot assistance condition. We used the data of the stationary period of gait for analysis. That is, we discarded the acceleration and deceleration phases in the first and end phases of gait, respectively. In addition, we removed the initial phase of the robot assistance from the data analysis to exclude the gait changing term by the assistance. For the Pre-, RwoA, and Post- conditions, we excluded the first and last three steps in each trial. For the RwA condition, we excluded three steps following the start of the rhythm assistance and the last three steps in each trial. For participants 6, 11, 14, and 15, the robot did not work properly in one trial under the RwA condition. Therefore, we excluded the trials from the calculation of the mean in the RwA condition. For participant 2, the robot did not work properly in two trials under the RwA condition. We then excluded the trials from the mean calculation. We used the Shaprio-Wilk normality test to confirm the normality of our data. To compare the mean values of the arm amplitude between the RwoA and RwA conditions, we performed the paired *t*-test. For the stride length, velocity, and CV of the stride duration, we used the one-way repeated ANOVA or the Friedman test when the normality of the data was confirmed or not, respectively. In the ANOVA, the sphericity of the data was checked using Greenhouse-Geisser method. If the sphericity was not confirmed, we adjusted the data by Greenhouse-Geisser's epsilon. For the *post-hoc* tests after the ANOVA and Friedman test, the Shaffer's method and multiple pairwise Wilcoxon signed-rank test using the Bonferroni correction (Bland and Altman, 1995) were conducted, respectively.

## Results

The means and standard deviations (SDs) between the participants for the arm swing amplitude were 37.9 ± 18.3 and 58.1 ± 23.7 (degrees) under the RwoA and RwA conditions. A Shapiro-Wilk normality test revealed the normality of the arm swing amplitude under the RwoA and RwA conditions (*p* = 0.082 and *p* = 0.380, respectively). For the swing amplitude, there was a significant difference between the RwoA and RwA conditions [*t*_(29)_ = 8.89, *p* < 0.001].

**Figure 4:**
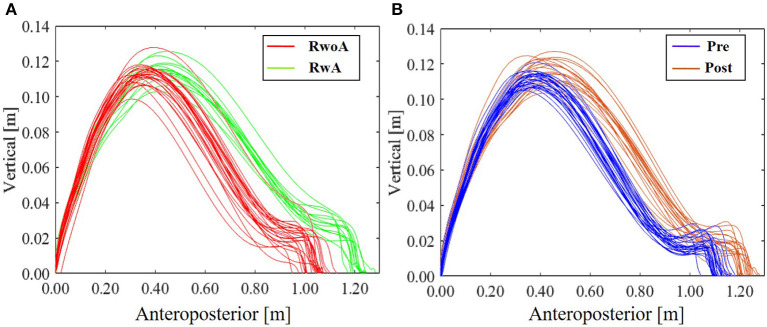
Sample shank trajectories under the four conditions. This sample trajectories were measured from the same participant. **(A)** RwoA and RwA conditions, **(B)** Pre- and Post-conditions. The error bars indicate the standard deviations between the patients.

**Figure 5:**
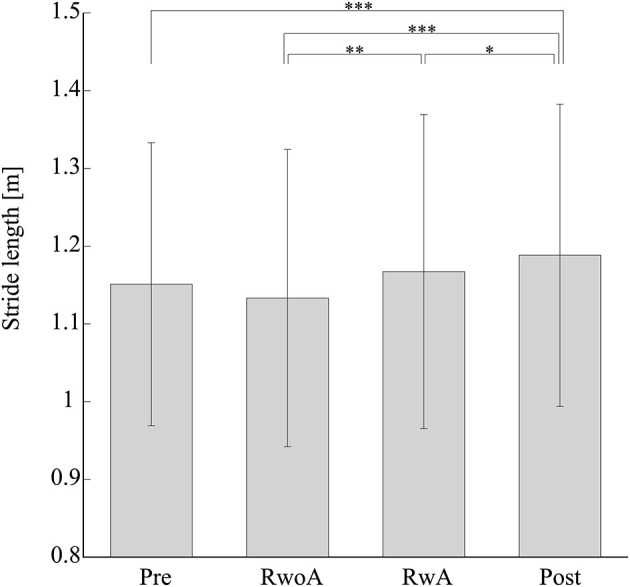
Means values of the stride length. *, **, and *** represent *p* < 0.05, *p* < 0.01, and *p* < 0.001, respectively. The error bars indicate the standard deviations between the patients.

**Figure 6:**
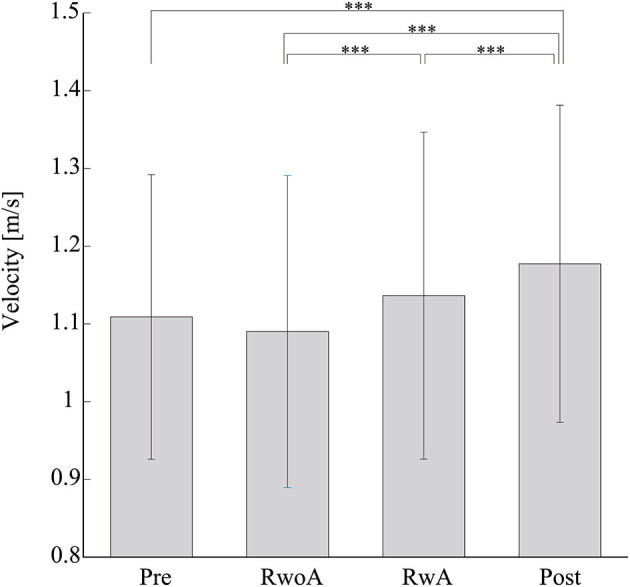
Mean values of the velocity. *** represents *p* < 0.001. The error bars indicate the standard deviations between the patients.

**Figure 7:**
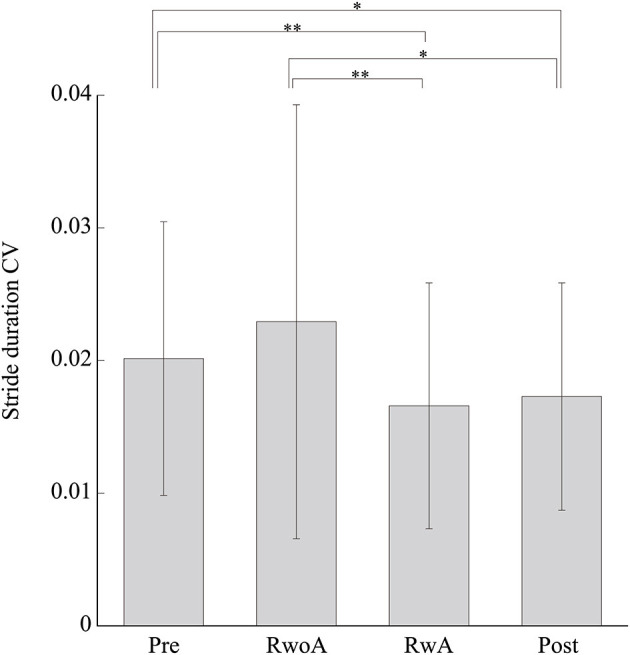
Mean values of the stride duration CVs. * and ** represent *p* < 0.05 and *p* < 0.01, respectively. The error bars indicate the standard deviations between the patients.

Figure 4 shows the samples of the shank trajectories in four conditions from the same participant. Comparing the RwoA and RwA conditions, the stride lengths under the RwA condition were larger than that under the RwoA condition (Figure 4A). Comparing the Pre- and Post-conditions, the stride lengths under the Post-condition were larger than that of the Pre-condition (Figure 4B). Figure 5 shows the mean values of the stride length, which were 1.15 ± 0.18, 1.13 ± 0.19, 1.17 ± 0.20, and 1.19 ± 0.19, respectively in the Pre, RwoA, RwA, and Post conditions, respectively. A Shaprio-Wilk normality test revealed the normality of the stride length data for the Pre, RwoA, RwA, and Post conditions (*p* = 0.506, *p* = 0.735, *p* = 0.723, and *p* = 0.994, respectively). A one-way repeated ANOVA revealed a significant difference between the four conditions [*F*_(2.42,70.12)_ = 13.97, *p* < 0.001]. The results of a *post-hoc* test show in Table 1. There was a significant difference between the RwoA and RwA conditions [*t*_(29)_ = 3.93, *p* = 0.001]. There was also a significant difference between the Pre and Post conditions [*t*_(29)_ = 4.90, *p* < 0.001]. Figure 6 depicts the mean values of the velocity. The means and SDs of the velocity between the participants were 1.11 ± 0.18, 1.09 ± 0.20, 1.14 ± 0.21, and 1.18 ± 0.20 in the Pre, RwoA, RwA, and Post conditions, respectively. A Shapiro-Wilk normality test showed the normality of the velocity data for the Pre, RwoA, RwA, and Post conditions (*p* = 0.731, *p* = 0.214, *p* = 0.293, and *p* = 0.565, respectively). A one-way repeated ANOVA revealed a significant difference between the four conditions [*F*_(2.39,69.40)_ = 25.43, *p* < 0.001]. A *post-hoc* test revealed a significant difference between the RwoA and RwA conditions [*t*_(29)_ = 4.44, *p* < 0.001]. Additionally, we found a significant difference between the Pre and Post conditions [*t*_(29)_ = 6.59, *p* < 0.001]. Figure 7 shows the mean values of the stride duration CV. The means and SDs of the stride duration CV between the participants were 0.020 ± 0.010, 0.023± 0.016, 0.017 ± 0.009, and 0.017 ± 0.009 in the Pre, RwoA, RwA, and Post conditions, respectively. A Shapiro-Wilk normality test did not reveal the normality of the stride duration CV for the Pre, RwoA, RwA, or Post conditions (all, *p* < 0.001). A Friedman test revealed a significant difference between the four conditions (χ^2^(3) = 17.4, *p* < 0.001). A *post-hoc* test revealed a significant difference between the RwoA and RwA conditions (*p* = 0.002). In addition, there was a significant difference between the Pre and Post conditions (*p* = 0.024).

**Table 1:** Results of the multiple comparison of the arm swing amplitude, stride length, velocity, and the stride duration CV.

	**Conditions**	**Difference**	***p***
Arm swing amplitude (degrees)	RwoA-RwA	−20.9	<0.001
Stride length (m)	Pre-RwoA	0.018	0.151
	Pre-RwA	−0.016	0.151
	Pre-Post	−0.037	<0.001
	RwoA-RwA	−0.034	0.001
	RwoA-Post	−0.055	<0.001
	RwA-Post	−0.021	0.014
Velocity (m/s)	Pre-RwoA	0.019	<0.001
	Pre-RwA	−0.027	0.121
	Pre-Post	−0.068	<0.001
	RwoA-RwA	−0.046	<0.001
	RwoA-Post	−0.087	<0.001
	RwA-Post	−0.046	<0.001
Stride duration CV	Pre-RwoA	−0.003	1.000
	Pre-RwA	0.004	<0.001
	Pre-Post	0.003	0.024
	RwoA-RwA	0.006	<0.001
	RwoA-Post	0.006	0.019
	RwA-Post	−0.001	1.000

**Figure 8:**
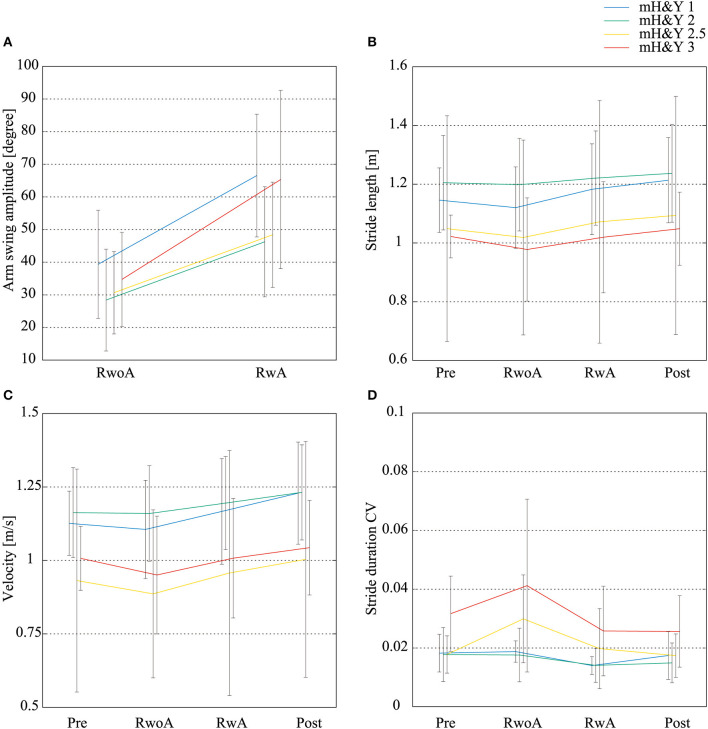
Mean values of the arm swing width, stride length, velocity, and stride duration CVs for each severity. **(A)** Arm swing amplitude, **(B)** stride length, **(C)** velocity, **(D)** stride duration CV. *N* = 4, 18, 3, and 5 for the patients with mH&Y 1, 2, 2.5, and 3, respectively. The error bars indicate the standard deviations between the patients.

Figure 8 shows the means of the arm swing amplitude, stride length, velocity, and stride duration CV for the patients with each severity. For all indices, the tendency of the difference between the conditions was similar to the means for all participants. That is, the arm swing amplitude in the RwA condition was increased compared to the RwoA condition for each severity. The stride lengths and velocity in the Post and RwA conditions were increased compared to the Pre and RwoA conditions, respectively. The stride duration CVs in the Post and RwA conditions were decreased compared to the Pre and RwoA conditions.

## Discussion

The purpose of this study was to verify the effect of upper-limb-rhythm assistance on the gait of patients with PD via synchronization between the patient's gait and the rhythm of the robot. We verified the two effects of a gait-assistance robot: the gait-assistance effect and after-effect. For the gait-assistance effect and after-effect, we compared the RwoA and RwA conditions, and Pre- and Post-conditions, respectively. The results revealed a gait-assistance effect and an after-effect. The arm swing amplitude, stride length, and velocity increased under the RwA condition compared to the RwoA condition. The stride duration CV decreased under the RwA condition compared to the RwoA condition. Additionally, the arm swing amplitude, stride length, and velocity were larger under the Post-condition than under the Pre-condition. The stride duration CV was smaller under the Post-condition than under the Pre-condition.

As one of the robot-assistance effects, the patient's arm swing was larger under the RwA condition than under the RwoA condition. Thus, the rhythm synchronization via tactile stimuli on the patient's upper limbs improved the patient's arm swing. The arm swing of the patients with PD was reduced (Lewek et al., 2010; Mirelman et al., 2016) and this weak arm swing led to an increased fall risk (Wood et al., 2002). Thus, an increase in the patients' arm swing using the proposed robot is expected to reduce their fall risk.

The robot was found to have a gait-assist effect on the lower-limb movements. The stride length and velocity increased under the RwA condition when compared to the RwoA condition. As mentioned above, there are neural couplings between the upper limbs and lower limbs (Huang and Ferris, 2009). In addition, periodic movements of the upper limbs increased the muscular activity of the lower limbs (Ferris et al., 2006; Kawashima et al., 2008). Thus, presenting a synchronizing tactile stimuli can influence patients' movements of the lower limbs via coupling between the upper and lower limbs. Although the velocity increase may increase the fall risk in the elderly (Van den Bogert et al., 2002), the stride duration CVs, which was one index for the gait stability and related to the fall risk (Schaafsma et al., 2003), was smaller in the RwA condition compared to the RwoA condition. These results suggest that using the robot assistance, the velocity would be improved while the fall risk decreased. Because fall risk assessment using data from wearable sensors is still challenging (Patel et al., 2020), investigation of whether rhythm assistance on the upper limbs decreases the risk of falls is required in the future. These effects on the lower limbs were shown in participants with all the severities (Figure 8). Thus, the robot assistance would improve the patient's lower limb movement with both mild and moderate severity. However, we could not conduct the statistical analysis for the results of each severity because of the small number of the patients for each severity. Thus, the effects of the proposed method on the gait of the patients with each severity should be investigated in a larger cohort of patients. These improvements in lower limb movements are the result of an enhancement of the central pattern generator (CPG) for gait. CPG includes specialized neural circuits in the spinal cord and generates the rhythmic movement for gait independently from the brain (Dimitrijevic et al., 1998; Yang et al., 1998). The coupling of cervical and thoraco-lumbar propriospinal systems coordinates the upper and lower limbs during walking (Dietz, 2002). The tactile stimuli on the upper limbs by the robot increased the arm swing amplitude, which increased the arm movement, enhancing CPG activation. As the result, the lower limb movement would be increased.

There were no significant differences between the Pre- and RwA conditions with regard to the stride length and velocity. Yap et al. revealed that the rhythm assistance provided by the robot increased the hip swing angle of healthy elderly participants when compared to pre-intervention (Yap et al., 2019). A reason for why we did not find a difference in the stride length and velocity between the Pre- and RwA conditions may be attributed to the small number of trials conducted. In a previous study (Yap et al., 2019), 15 trials under the robot-rhythm-assist conditions were conducted for each participant. In contrast, this study only performed three trials under the robot-rhythm-assist condition for each patient.

The stride duration CV was smaller under the RwA condition than under the RwoA condition. Thus, this result revealed the assist-effect of the proposed robot on gait stability. In addition, the stride duration CV was smaller under the RwA condition than under the Pre-condition. Thus, this effect on the stride duration CV would be higher than the stride length and the velocity. These results revealed that the synchronized tactile stimulation increased gait stability in PD patients. Hove et al. pointed out that the 1/*f*-like structure in the stride duration could increase the gait stability (Hove et al., 2012). The 1/*f* –like structure makes the time series more predictable, which would increase the perceived movement stability. In fact, the fractal scaling is an index of gait stability (Hausdorff, 2009). In our experiment, the synchronized tactile stimulation on the upper limbs would reinstate the 1/*f*-like structure in the stride duration as is the case with synchronized auditory stimulation. Thus, the stride duration CV decreased in the RwA condition. Note that we could not calculate the fractal scaling in this study as a much longer time series is needed; however, it is impossible for the participants to walk such a long distance whilst wearing the robot. Future work should investigate the effect of the robot assistance on the fractal scaling of the stride duration.

We found the after-effects with regard to the stride length, velocity, and stride duration CV. The stride length and velocity were larger under the Post-condition than those under the Pre-condition. In addition, the stride duration CV was smaller under the Post-condition than that under the Pre-condition. It was reported that the stride length and velocity was related to the risk of freezing of gait (Nieuwboer et al., 2001; Chee et al., 2009; Contreras and Grandas, 2012). Therefore, gait training by providing rhythm assistance on the upper limbs could reduce the risk of freezing of gait. In addition, one of the gait disorders found in patients with PD is hypokinesia of the lower limbs (Morris et al., 1994; Hass et al., 2012). The after-effect of increasing stride length and velocity by the proposed robot is expected to improve lower limb hypokinesia. Additionally, in patients with PD, the stride duration CV was higher than that of healthy elderly participants (Weiss et al., 2011), and it was related to fall risk (Schaafsma et al., 2003). Thus, the after-effect of the stride duration CV may lead to a reduction in fall risk.

It is important to note that the after-effect observed in this study included the effect of gait under the RwoA condition. The reason we applied this condition was to observe the effect of wearing the robot. As a result, there were no significant difference between the Pre- and RwoA conditions for the stride length, velocity, and stride duration CV, suggesting there was no influence from wearing the robot. In future work, it is necessary to verify the after-effect when excluding the effect of gait while wearing the robot without rhythm assistance.

We did not observe any significant differences between the Pre and RwoA conditions for the stride length, velocity, and stride duration CV (Table 1 and Figures 5–7). This result suggests that the weight of the robot did not affect the patient's gait across all participants nor did it increase the fall risk. However, the robot weight might decrease the gait performance of the patients, especially those with a relatively high severity. The stride length and velocity in the RwoA conditions were lower than those in the Pre conditions for the patients with mH&Y 2, 2.5, and 3. In addition, the stride duration CV in the RwoA conditions was higher than that in the Pre-condition for mH&Y 2.5 and 3. Thus, the weight of the robot might be heavy for these patients; however, it is not yet clear because of the small sample size for the patients with each severity. The decrease in gait performance would increase fall risk (Schaafsma et al., 2003) and decrease or cancel out the effects of the robot assistance. In future work, weight reduction of the robot is required.

There are some limitations in this study. The values of the parameters in the system, α, β, *k*_*hm*_, *k*_*m*_*rl*_, θ_*d*_, and motor torque were determined by trial and error. Thus, more efficient values for each parameter should be investigated in future work. In addition, these parameters might be different between individuals. Therefore, the method to determine the values suitable for individuals should be constructed. For patient safety, the experimenters walked behind the patients in the trials. Thus, the patients might hear the footsteps of the experimenter, thus the influence of auditory signals has not been completely eliminated in this experiment. In addition, we could not omit the order effect. Although we could not perform more trials due to patient burden, in the future, the repetition effect should be considered using another experimental design such as the A-B-A-B design. In addition, although we conducted three trials for the RwA conditions, the number of the trials should be the same between all conditions in the future.

We found that the rhythm synchronization between the gait of the PD patients and the tactile stimuli on their upper limbs statistically changed their gait. In future studies, it is necessary to verify how the rhythm synchronization improved symptoms in the gait of the patients with PD, as the cause of gait ability decline in patients with PD is different from that in healthy elderly individuals (Rocha et al., 2014). The appearance of symptoms in gait depends on the severity of the condition and each individual. Therefore, it is important to investigate the relationship between the gait symptoms of each participant and the assistance by the proposed robot in a large number of patients with PD. In addition, study of the long-term after-effects and/or intervention for patients with PD using the robot is also required. Although the after-effects observed in this study, with regard to stride length, velocity, and stride duration CV, were just immediate effects after gait with the robot assistance device, these results show the possibility of using the proposed robotic device in gait rehabilitation for patients with PD. Furthermore, an improvement of patient's gait function should be investigated from a medical viewpoint using standard clinical tests such as the 10 m walk and dynamic gait indexes.

## Conclusion

The purpose of this study was to investigate whether the rhythm-assist robot changed the gait of the patients with PD. The robot produced tactile stimuli on the patient's upper limbs, which synchronized with the arm swing of the patients. First, we verified the gait assistance effect by the proposed robot. The arm swing amplitude, stride length, and velocity were increased in the gait with rhythm-assistance provided by the robot when compared to the gait when wearing the robot without assistance. In addition, the stride duration CV was significantly decreased in the gait with the robot's rhythm assistance compared to the gait with wearing the robot without assistance. Secondly, we found immediate after-effects of the robot's rhythm assistance. The stride length and velocity were increased after the robot intervention when compared to the pre-intervention. Additionally, the stride duration CV was decreased in the post-intervention when compared to the pre-intervention. These results suggest that the rhythm assistance provided by the robot may play a role in gait rehabilitation for patients with PD.

## Data Availability Statement

The datasets for this article are not publicly available because Ethics Committee does not allow the disclosure of patient data. Requests to access the datasets should be directed to Taiki Ogata, ogata@c.citech.ac.jp.

## Ethics Statement

This experiment was conducted with the approval from Tokyo Institute of Technology's Research Ethics Review Committee and Kanto Central Hospital Ethics Committee. Informed consent was obtained in writing from all participants.

## Author Contributions

TK, TO, RS, MN, and MS conducted the experiments. SO recruited the participants. TK and RS analyzed the data. All authors interpreted the results of the analyses. TK drafted the manuscript. All authors gave approval of the final version of the manuscript to be published.

### Conflict of Interest

MN was employed by Kikuchi Seisakusho Co. Ltd. and MS was employed by WALK-MATE LAB Co. Ltd. The remaining authors declare that the research was conducted in the absence of any commercial or financial relationships that could be construed as a potential conflict of interest.
